# A cancer-associated *TP53* synonymous mutation induces synthesis of the p53 isoform p53/47

**DOI:** 10.1038/s41416-025-03127-w

**Published:** 2025-07-26

**Authors:** Rhythm Sajwan, Lixiao Wang, Olivera Casar-Borota, Konstantinos Karakostis, Sa Chen, Robin Fahraeus, Xiaolian Gu, Sivakumar Vadivel Gnanasundram

**Affiliations:** 1 Department of Medical Biosciences, Umea University, Umea, Sweden; 2 Department of Immunology, Genetics and Pathology, Uppsala University, Uppsala, Sweden; 3 Research Programme on Biomedical Informatics (GRIB), Hospital del Mar Research Institute (PRBB), Barcelona, Spain; 4 Biochemistry and Molecular Biology Department, University of Valencia, Valencia, Spain; 5 Inserm U1131, 27 Rue Juliette Dodu, Paris, France; 6 RECAMO, Masaryk Memorial Cancer Institute, Brno, Czech Republic; 7 Department of Biological Sciences & Engineering, Indian Institute of Technology Palakkad, Palakkad, India

**Keywords:** Tumour-suppressor proteins, RNA

## Abstract

**Background:**

Synonymous mutations (SMs) change the mRNA nucleotide sequences without altering the corresponding amino acid sequence and are usually overlooked due to their perceived lack of influence on protein function. However, emerging reports suggest that SMs play a significant role in disease development and progression.

**Methods:**

Whole exome sequencing, RNA-sequencing, and droplet digital PCR were performed to identify the SMs from the malignant glioma patients. MutaRNA was used to predict the effect of SMs on RNA structure in silico. SHAPE-MaP was performed to probe and assess the effect of SMs on RNA structure in-cellulo.

**Results:**

Here, we report that a Cancer-Associated SM in *TP53* codon valine 203 (CASM203) results in the induction of the alternative translation initiated p53 protein isoform, p47. In-cell high-throughput RNA structural mapping showed that *CASM203* mimics the Protein Kinase RNA-Like ER Kinase (PERK)-mediated *p53* mRNA secondary structure that induces p47 expression of during the unfolded protein response (UPR).

**Conclusions:**

Overall, the single gain-of-function SM mimics the UPR-mediated p53 stress response, by generating RNA secondary structures akin to the PERK-mediated *p53* mRNA structural switch. This illustrates the link between RNA structures and cellular biology and underscores the importance of SMs in cancer biology and their potential to further refine genetic diagnostics.

## Background

The sequential accumulation of mutations that affect expression or function of the encoded proteins is a hallmark of cancer. Synonymous mutations (SMs) change RNA sequences but not the corresponding amino acid sequences due to redundancy in the genetic code. SMs are usually interpreted as silent and are not included in current personalized molecular diagnostic panels. However, emerging reports indicate that SMs may affect the function of the encoded protein and are associated with numerous human diseases, including cancers, displaying a similar probability of disease-association as other somatic mutations [1–4]. SMs are reported to represent about 8% of all single-nucleotide variant driver mutations in oncogenes [3, 5], and they are also predicted to have potential gain-of-function in cancer-associated genes [6]. Apart from affecting splicing [7, 8], SMs can alter mRNA stability and interactions with miRNAs and RNA binding proteins. The implication of SMs on RNA structural changes are less studied, with only a few examples from *CFTR, KRAS*, and tumor suppressor *TP53* mRNAs [4, 9, 10].

Nonsense or somatic loss-of-function missense mutations in the *TP53* gene that prevent p53 from carrying out its tumor suppressor activity are observed in more than half of human cancers of diverse cellular origins [11]. In addition, multiple studies show that missense gain-of-function (GOF) *TP53* mutations can promote a malignant tumor phenotype [12]. *TP53* SMs constitute about 6% of total cancer-associated *TP53* mutations but are rarely investigated, partly due to the lack of information regarding the mechanism(s) by which mRNAs may affect the function of the encoded protein [8]. However, previous work from our group has shown that a cancer-associated SM in *TP53* codon 22 (CASM22) prevents p53 induction during the DNA damage response by altering protein-mRNA interactions [13].

The human p53 isoform, p47 (also called p53/47 or Δ40p53) lacks the first 39 amino acids and is derived from alternative initiation of translation at the 2^nd^ in-frame AUG at codon 40 via structural changes in the human *p53* mRNA, following activation of the PERK during the UPR [14, 15]. p47 potentially forms homo- or hetero-oligomers with p53 and exhibits a functional diversity, including G2 arrest via induction of *14-3-3σ*. p47 is detected in damaged neural tissue and in glioblastoma, and the corresponding murine isoform (p44) causes premature ageing and altered stem cell pluripotency [16–18].

Here, we report that mRNA derived from the *TP53* CASM at codon 203 (CASM203), seen in human glioblastoma and ovarian carcinoma, shows a similar RNA secondary structure to that seen during UPR and exhibits de novo gain-of-function activity by inducing p47.

## Materials and methods

### Cell culture, transfection, and treatments

H1299 p53-null cells (non-small-cell lung carcinoma human cell line; NCI-H1299 (ATCC CRL-5803)) was mostly used for experimental analysis, unless specified otherwise. Other cell lines used were HCT116 p53-null cells (human colon cancer cell line (CVCL_HD97)) and A375 p53-null cells (Human melanoma cell line (ATCC CRL-1619)) [19]. Cell lines were routinely cultured in RPMI 1640 medium (31870074, Thermo Fisher Scientific; for H1299 p53-null cells) or in DMEM–Dulbecco’s Modified Eagle Medium (11960069, Thermo Fisher Scientific; for A375 p53-null cells) or in McCoy’s 5a Medium (M8403, Sigma Aldrich; for HCT116 p53-null cells) supplemented with 10% fetal bovine serum (A3160502, Thermo Fisher Scientific), 100 U.ml^−1^ penicillin and 100 mg.ml^−1^ streptomycin (15140122, Thermo Fisher Scientific) and 2 mM L-glutamine (25030081, Thermo Fisher Scientific) and maintained at 37 °C in a humidified 5% CO^2^ incubator. Cells are routinely checked for mycoplasma contamination using MycoStrip™ - Mycoplasma Detection Kit (rep-mys-10, Invivogen). Plasmid DNA transfections were performed using GeneJuice reagent (70967, Sigma-Aldrich) following the manufacturer’s protocol. ER stress was induced by treating cells with 100 nM thapsigargin (Thap) (T7459, Thermo Fisher Scientific) in dimethyl sulfoxide (DMSO) (276855, Sigma-Aldrich) for 16 h. DMSO alone was used as a control.

### Plasmid constructs

cDNA constructs used in the study were generated using the pcDNA3 eukaryotic expression vector (Life Technologies, Carlsbad, CA, USA). p53-WT construct has been described previously [15]. Synonymous mutations inserted into the p53 coding sequences (CASM203/ c.G609T, c.G609A, and c.G609C) were carried out using site-directed mutagenesis. Plasmid constructs generated for this study are deposited in Addgene (IDs: 229531; 229532; 229533).

### DNA isolation from glioma tissues and whole exome sequencing (WES)

Tissue samples (*n* = 42) from patients diagnosed with glioma were collected through Uppsala Biobank and U-CAN (www.u-can.uu.se), Sweden [20]. Ten oligodendrogliomas (3 WHO grade 2 and 7 WHO grade 3), 11 *IDH* mutant astrocytomas (6 WHO grade 2, 3 WHO grade 3 and 2 WHO grade 4) and 11 *IDH*-WT glioblastomas were included. The study was approved by the Swedish Ethical Review Authority (Dnr 2019-01595) and performed in accordance with the Declaration of Helsinki. All tissue biopsies were rapidly frozen in dry ice and isopentane upon arrival at the pathology department and stored at −80 °C until extraction of nucleic acids. An experienced neuropathologist (OC-B) reviewed frozen sections from all glioma specimens to confirm the presence of representative tumor tissue and assess the proportions of tumor cells. Genomic DNAs were isolated from the homogenized glioma tissue samples using Allprep DNA/RNA micro kit (Qiagen, 80284) following the manufacturer’s instructions. Isolated DNAs were checked for sample quality and pursued for whole exome sequencing (WES) using the Agilent SureSelect kit v6 (Agilent Technologies). Following library preparation, sequencing was performed with the pair-end 150 (PE150) strategy using Illumina NovaSeq 6000 platform (Novogene, UK). The WES data were analyzed using the nf-core sarek pipeline (https://nf-co.re/sarek/3.4.4/) to call somatic variants, which were then annotated with SnpEff.

### Droplet digital PCR (ddPCR)

The WES genotyping results of CASM203 (chr17: 7674922G>T) were validated by quantitative ddPCR amplification in aqueous droplets within an oil phase (emulsion PCR) using the QX200 ddPCR system (Bio-Rad) and EvaGreen chemistry (ddPCR™ Supermix, Biorad #1864033) following the manufacturer’s instructions. The following oligonucleotides were used: p53_Ex6 5′-CATGAGCGCTGCTCAGATA-3′ was used as forward primer, and either p53_609_G 5′-GTGTTTCTGTCATCCAAATACTCC-3′ or p53_609_T 5′-GTGTTTCTGTCATCCAAATACTCA-3′ were used as reverse primers to amplify the G609 (WT) or the T609 (CASM203) variant, respectively. All primers and probes were tested in duplex experiments in 96-well plates in total volume of 22 μL. Sample DNA #25 was tested, with the pcDNA3_p53-G609T construct used as a positive control (p53_609T) and DNA extracted from the human lymphoblastoid cell line (NA12156) as a negative control (p53_609G). PCR negative control with no template was used for normalization. 25 fg of plasmid DNA or 25 ng of genomic DNA were used as templates for normalizing the copy number of the gene in the input.

### Western blotting

Cell lysates were prepared using RIPA buffer (Thermo Fisher Scientific) supplemented with complete protease inhibitor cocktail (Roche, Basel, Switzerland). Equal protein amounts were resolved in SDS-PAGE and blotted. Immunoblots were probed with anti-p53 (CM-1-Recamo; # 9282, Cell Signaling Technology) and anti-actin (AC-15, Sigma-Aldrich), and detection was performed using WestDura (Thermo Fisher Scientific) with ChemiDoc™ Touch Imaging System (Bio-Rad). Western blots represent *n* ≥ 3.

### RNA isolation and RT-qPCR

Total RNA was purified from H1299 cells post-transfection using the RNeasy Mini Kit (74104, Qiagen) following the manufacturer’s protocol. RT was carried out using Superscript II Reverse Transcriptase (18064014, ThermoFisher Scientific) and oligo(dT) primers (18418012, ThermoFisher Scientific). qPCR was performed on QuantStudio™ real-time PCR system (Applied Biosystems) using PowerUp™ SYBR™ Green Master Mix (A25741, ThermoFisher Scientific) with the corresponding target primer sets. Primer sequences: *TP53*, forward 5′- GTCTGGGCTTCTTGCATTCTG-3′; reverse 5′-GCTGTGACTGCTTGTAGATGGC-3′. *SFN* (14-3-3σ) forward 5′-TGCTGGACAGCCACCTCATCAA-3′; reverse 5′-GGCTGAGTCAATGATGCGCTTC-3′. *ACTB*, forward 5′-TCACCCACACTGTGCCCATCTACGA-3′; reverse 5′-TGAGGTAGTCAGTCAGGTCCCG-3′. *CDKN1A* (p21) forward 5′-CCTCAAATCGTCCAGCGACCTT-3′; reverse 5′-CATTGTGGGAGGAGCTGTGAAA-3′. *PMAIP1* (NOXA) forward 5′-TAAAGCAAGAATGGAAGAC-3′; reverse 5′-GACCGAAGAAATCAACAC-3′.

### In-cell RNA SHAPE-MaP

SHAPE-MaP was performed as described previously [15, 21]. Briefly, H1299 cells grown in 6 well plates were transiently transfected with the indicated constructs. 36 h post-transfection, cells were washed with PBS and 900 ul of RPMI media was added. The SHAPE reagent 1-Methyl-7-nitroisatoic anhydride (1M7) (Sigma-Aldrich) was added to a final concentration of 10 mM by adding 100 µL of 100 mM 1M7, and cells were treated for ~90 seconds at 37 °C. The same volume of DMSO was added to the untreated samples. Cells were washed with PBS and harvested. RNA purification was carried out using the RNeasy kit (Qiagen), followed by DNAse I digestion for 30 min at 37 °C. Reverse-transcription of purified RNA was carried out with the *TP53* primer (5′-TAGTTGTAGTGGATGGTGGTACA-3′) using MaP buffer and Superscript II Reverse Transcriptase (Thermo Fisher Scientific). Synthesized cDNAs were then purified and amplified using Q5 DNA polymerase (NEB) using p53 primers (forward 5′- ATGGAGGAGCCGCAGTCAGAT-3′; reverse 5′-TAGTTGTAGTGGATGGTGGTACA-3′). The PCR product was purified and quantified with a Qubit fluorometer and diluted to 0.2 ng/µl. Purified amplicons were then tagmented and a library was created using the Ilumina Nextera PCR library kit. Library products were purified with Agencourt AMPure XP beads (Beckman Coulter Life Sciences) as described [22]. The library concentration was measured with a Qubit fluorometer and the size distribution assessed using an Agilent 2100 Bioanalyzer. Libraries were then sequenced with NovaSeq PE150. SHAPE reactivity profiles and comparisons were generated using ShapeMapper 2 with default settings [22] and aligned to p53 coding sequences. SHAPE-Map data shown are representative of at least two independent biological repeats.

### Statistical analysis

Statistical significance was analyzed by comparing data sets with corresponding reference points using Student’s unpaired *t* test (**p  <  0.001; **p  <  0.01; *p  <  0.05; ns-not significant). For ddPCR data, two-tailed t-test was employed to calculate significance (****p  <  0.0001; ***p  <  0.001; **p  <  0.01; *p  <  0.05; ns-not significant). Statistical assessments were performed using GraphPad Prism software.

## Results and discussion

### A cancer-associated synonymous TP53 mutation promotes p47 isoform expression

**Fig. 1:**
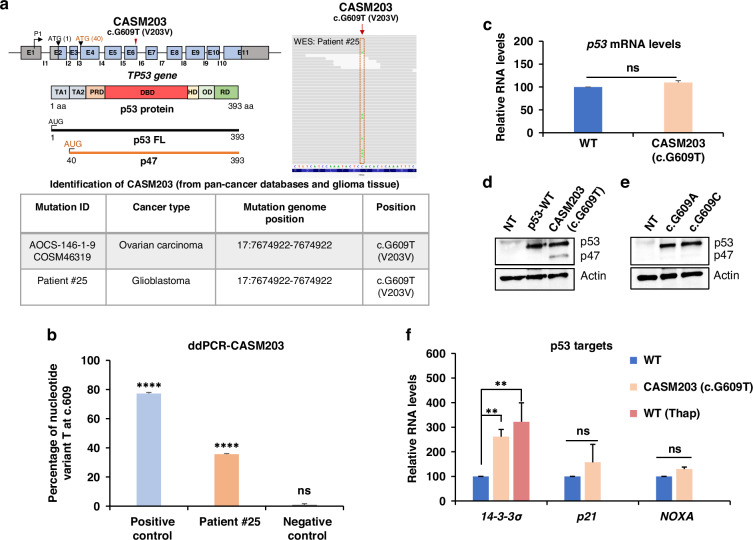
CASM203 induces p47 isoform translation and activates *14-3-3σ.* **a** Schematics showing the *Tp53* gene architecture, exons and introns are numbered according to canonical annotation; position of CASM203 is indicated in exon 6. Underneath, functional domains of p53 full-length (FL) protein and p47 isoform are illustrated, transactivation domains (TAs); polyproline domain (PRD); DNA binding domain (DBD); hinge domain (HD); the oligomerization domain (OD); the C-terminal regulatory domain (RD). Positions of 1^st^ AUG and 2^nd^ AUG (40 aa) which is involved in the p47 isoform translation are indicated, p47 isoform lacks the transactivation domain 1. The side panel shows snapshot of CASM203 identification from the WES of patient #25. Lower panel: Table showing the details of CASM203 identification. **b** Bar-graph showing the confirmation of CASM203 (c.G609T) by droplet digital PCR (ddPCR). Normalized ratios of mutant variant T at c.609 were plotted. Plasmid bearing the *TP53*-G609T mutation was used as positive control and genomic DNA extracted from the human lymphoblastoid cell line was used as a negative control. Mean ± s.d of three independent replicates were plotted (****p  <  0.0001; ***p  <  0.001; **p  <  0.01; *p  <  0.05; ns-not significant). **c** Bar graph comparing *TP53*-WT and CASM203 transcript levels following expression in p53 null H1299 cells. **d** Western blot showing induction of the p47 isoform by CASM203 (c.G609T) under normal conditions in H1299 cells. **e** Other codons at valine 203 (c.G609A, c.G609C) do not affect p47 expression. NT-mock control, cells transfected with empty vector. Actin was used as a loading control. **f** Bar graphs showing the relative transcript levels of p53 downstream targets (*p21, 14-3-3σ*, and *NOXA*) in H1299 cells expressing p53-WT and CASM203. For *14-3-3σ* p53-WT treated with Thap was also plotted. The RT-qPCR graphs are plotted as mean ± s.d of three independent experiments. Statistical significance was calculated using unpaired t-test (***p  <  0.001; **p  <  0.01; *p  <  0.05; ns-not significant).

*TP53* SMs from the Synonymous Mutations In Cancer database (SynMICdb) [4] were screened in silico for potentially altering cellular function and RNA structures. The *TP53* c.G609T SM at codon valine 203 (CASM203) from ovarian carcinoma, showed a good RNA structure change and SynMICdb scores, reflecting a strong likelihood of impacting the cellular function. We also identified CASM203 (c.G609T) via WES in a glioblastoma tissue sample (patient #25) (Fig. 1a), and we confirmed the WES results by performing droplet digital PCR (ddPCR) using the nucleotide variant oligos at position c.609 (G/T). In line with the WES data, ddPCR results showed that the tumor sample of patient #25 has a heterogenous genotype. The normalized ratio of copies carrying the mutated nucleotide (T) to the non-mutated (G) was T (35%): G (65%), revealing the heterozygosity. The ratio of the negative control (gDNA extracted from NA12156) was T (0%): G (100%) and of the positive control (plasmid construct bearing p53-G609T mutant) was T (76%): G (24%) (Fig. 1b).

When CASM203 was expressed in p53-null H1299 lung carcinoma cells, we observed no significant difference in *TP53* mRNA levels, or full-length p53 protein levels, compared to *TP53*-WT (Fig. 1c). However, we observed a significant induction of p47 in cells expressing CASM203 under normal conditions (Fig. 1d). Valine is encoded by four codons (GTG, GTT, GTA, and GTC) and when we introduced the c.G609A and c.G609C codons at position 203, we did not observe the induced p47 expression, demonstrating the specificity by which the CASM203 (c.G609T) controls p47 expression (Fig. 1e). Similarly, CASM203 induced p47 expression also in A375 and HCT116 p53-null cancer cells (Supplementary Fig. 1a, b), illustrating that CASM203 consistently induces p47 expression in multiple p53-null cell lines derived from different cancers. p47 expression from *p53*-WT mRNA following PERK activation results in downstream activation of *14-3-3σ* and G2 cell cycle arrest [14, 23]. Expression of p47 from CASM203 in the absence of PERK activation showed an increase in *14-3-3σ* mRNA levels whereas other p53-dependent targets such as *p21* and *NOXA* were not significantly altered (Fig. 1f). Together, these results show that a single patient-derived nucleotide change downstream of the p47 initiation site is sufficient to change translation initiation of the *p53* mRNA and mimic the UPR-mediated p53 response and 14-3-3σ activation. They also underline that *p53* mRNA structural changes are sufficient for the UPR-mediated p53 stress response.

Numerous studies have shown that the UPR pathway promotes various aspects of tumor progression and chemo resistance and is active in a wide range of cancers [24]. CASM203 mimicking the UPR-mediated p53 stress response raises the possibility that CASM203 plays an active role in the tumorigenesis of some cancers and that it is acquired due to selection. In support of this, the two other codon changes of valine 203 had no effect on p47 expression. Transgenic animals expressing the murine equivalent of p47, p44, demonstrate severe pre-mature ageing phenotypes and altered stem cell pluripotency in a p53-dependent fashion, indicating that changing the ratio of p53:p47 has an important impact on cell physiology [16–18].

### CASM203 mimics the UPR-mediated changes in the p53 mRNA structure

**Fig. 2:**
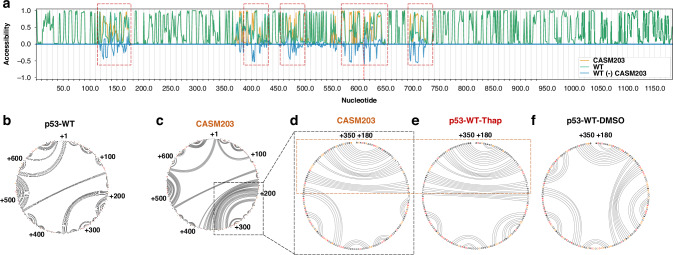
CASM203 alters *p53* mRNA structure similar to PERK-mediated *p53* mRNA folding. **a** The accessibility profiles (based on in silico predictions using mutaRNA web server [25]) of p53-WT (green) and CASM203 (orange) and their differences (blue) show the mutation’s effect on the RNA’s single-strandedness, accessibility is measured based on local single-position unpaired probabilities. The mutated position is highlighted by a red line. Circos plots showing the base-pairing probabilities based on the SHAPE reactivity of p53-WT (**b**) and CASM203 (**c**). Numerous changes in base-pairing probabilities were observed in CASM203 compared to WT. SHAPE-modified nucleotide sequences are indicated in orange/red. Circos plots showing the comparisons of base-pairing probabilities of p53-CDS regions +180 to +350 nts between (**d**) CASM203, (**e**) p53-WT-Thap (UPR), and (**f**) p53-WT-DMSO (for p53-WT-DMSO and Thap, SHAPE reactivities are extracted from ref. [15]). Regions of CASM203 display structural similarities induced by Thap are boxed and highlighted. **d**–**f** regions from +180 to +350 are zoomed in and indicated with a box.

PERK-mediated folding of the *p53* mRNA during activation of the UPR promotes alternative translation initiation, leading to the production of the p47 isoform independently of eIF2α [15]. Since CASM203 was found to induce p47 isoform expression, we sought to determine whether it promotes a similar mRNA folding pattern as observed during the UPR. To investigate this, we first performed an in silico analysis using the MutaRNA web server, which predicts and visualizes mutation-induced alterations in RNA secondary structure [25]. The analysis revealed that CASM203 modifies absolute base-pairing probabilities and RNA accessibility at multiple sites within the *p53* mRNA (Supplementary Fig. 2), including both the vicinity of the mutation and distal regions. Notably, we observed changes in RNA accessibility within the region spanning +180 to +350 nucleotides—an area previously shown to mediate p47 induction during UPR (Fig. 2a).

To experimentally validate the structural impact of CASM203 on *p53* mRNA, we employed SHAPE-MaP to probe RNA structure in cells. This was conducted in H1299 cells expressing either wild-type p53 (p53-WT) or the CASM203 variant. Compared to p53-WT, CASM203 expression resulted in multiple changes in base-pairing probabilities across the TP53 coding sequence. Secondary structures predicted by Superfold, based on SHAPE reactivity profiles, revealed significant structural rearrangements in CASM203 relative to WT, particularly around the second AUG codon responsible for p47 isoform translation, as well as in downstream regions of CDS (Fig. 2b, c and Supplementary Fig. 3a, b). Arc plots indicated that these base-pairing changes observed in corresponding circus plots are statistically significant (Supplementary Fig. 4a, b). Importantly, structural changes were also observed in the +180 to +350 region of the p53 coding sequence, a region known to mediate p47 isoform expression during UPR, reinforcing the similarity between CASM203-driven and UPR-driven *p53* mRNA folding.

**Fig. 3:**
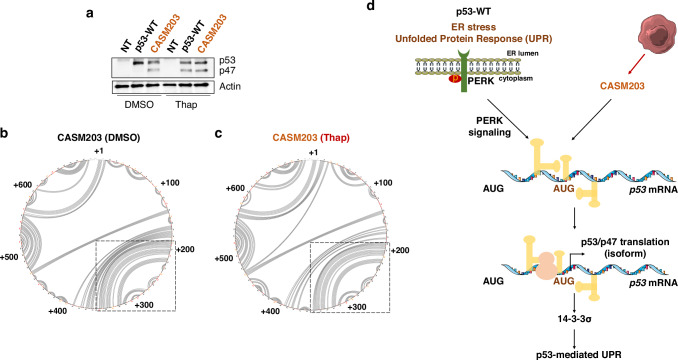
CASM203 mimics UPR-mediated p53 stress response. **a** Western blot showing p47 isoform under normal and UPR conditions (Thap) in H1299 cells expressing p53-WT or CASM203. p53-WT showed the induction of p47 isoform only upon UPR. Circos plots showing the base-pairing probabilities of CASM203 under normal (**b**) and UPR conditions (Thap) (**c**). CASM203 does not undergo a PERK-mediated RNA structural switch (indicated as a dashed box) with Thap treatment. **d** Model showing the de novo gain-of-function effect of cancer-associated *TP53* synonymous mutation CASM203. During the UPR, *p53-WT* mRNA undergoes PERK mediated RNA folding to initiate p47 translation and thereby stimulate *14-3-3σ* and cause G2-cell cycle arrest. CASM203 generates a *p53* mRNA secondary structure similar to that promoted by PERK and thereby mimics UPR-mediated p53 stress response.

Treatment with endoplasmic reticulum (ER) stress-inducing agents such as thapsigargin (Thap) or tunicamycin activates the UPR. Comparative analysis of secondary RNA structures spanning nucleotides +180 to +350 revealed that the *CASM203* RNA structure under basal conditions closely resembled the *p53-WT* mRNA structure during UPR activation following treatment with 1 µM Thap (Fig. 2d–f and Supplementary Fig 5a–c). Importantly, no further increase in expression p47 was observed in CASM203-expressing cells following Thap treatment (Fig. 3a). Consistent with this, SHAPE-MaP analysis showed no additional structural changes in *CASM203* RNA following Thap treatment, indicating that the mutation renders the RNA structure pre-configured in a UPR-like conformation even under non-stressed conditions (Fig. 3b, c). Arc plots show that the base-pairing probabilities observed in corresponding circular plots in Fig. 3b, c are significant (Supplementary Fig. 4c, d). Corresponding SHAPE reactivity of profiles of these datasets are shown in Supplementary Fig. 6a, b. The 5′ untranslated region (UTR) of p53 neither affected the structural impact of CASM203 on *p53* mRNA nor influenced the induction of the p47 isoform (Supplementary Figs. 7, 8).

We have previously shown that a cancer-associated *TP53* synonymous mutation in codon 22 targets *p53* RNA structure and prevents expression of the full-length p53 protein following DNA damage [9, 13]. Together with the results presented here, this shows that CASMs in *TP53* may have different effects on the translation of *p53* mRNA that either mimic, or interfere, with signaling response pathways. This highlights the importance of RNA structures in the cellular response to stress conditions and demonstrates how SMs may interfere with the formation of these structures. Moreover, it is interesting to note that both CASM22 and CASM203 are within the coding sequence, illustrating how p53 has exploited the degenerative genetic code to allow RNA structures to control the activity of the encoded protein in response to various signaling pathways while the protein has evolved in parallel to induce suitable cell biological effects in response to various cellular stress pathways.

## Conclusion

Altogether, our data demonstrates that the cancer patient-derived de novo gain-of-function *TP53* synonymous mutation CASM203 resembles the *p53* mRNA structure induced by the UPR (Fig. 3d). The fact that a single nucleotide SM mimics a natural mechanism involved in stress response by imposing alternative translation initiation and protein isoform synthesis highlights the sophisticated dynamics of mRNA structures and their relevance in cellular stress response pathways.

## Supplementary information

Supplementary figures

Supplementary information_raw western blots

## Data Availability

The authors declare that data supporting the findings of this study are available within the article and/or in the supplementary information. SHAPE-MaP raw data are available at https://github.com/medbioumu/suppldata_casm203.
