# Supplementary material for: A cancer-associated TP53 synonymous mutation induces synthesis of the p53 isoform p53/47
Source: Br J Cancer. 2025 Jul 26;133(7):970–5. doi: 10.1038/s41416-025-03127-w (PMC12480914; doi:10.1038/s41416-025-03127-w)
Supplement: Supplementary file 1 — Supplementary figures [file 41416_2025_3127_MOESM1_ESM.pptx]

## Slide 1
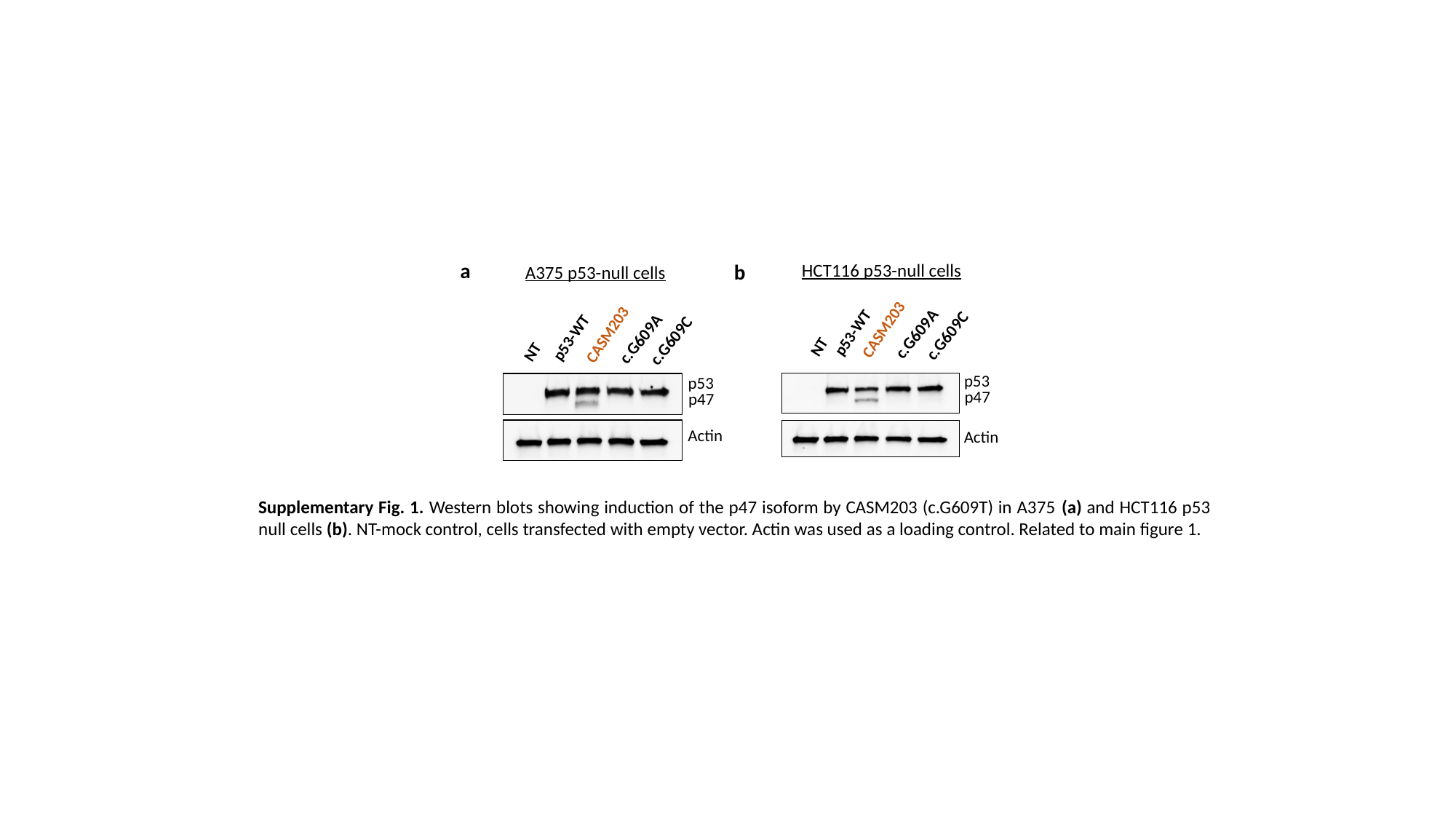

CASM203
c.G609A
NT
c.G609C
p53
p47
Actin
p53-WT
p53-WT
c.G609A
NT
c.G609C
p53
p47
Actin
CASM203
a
b
HCT116 p53-null cells
A375 p53-null cells
Supplementary Fig. 1. Western blots showing induction of the p47 isoform by CASM203 (c.G609T) in A375 (a) and HCT116 p53 null cells (b). NT-mock control, cells transfected with empty vector. Actin was used as a loading control. Related to main figure 1.

## Slide 2
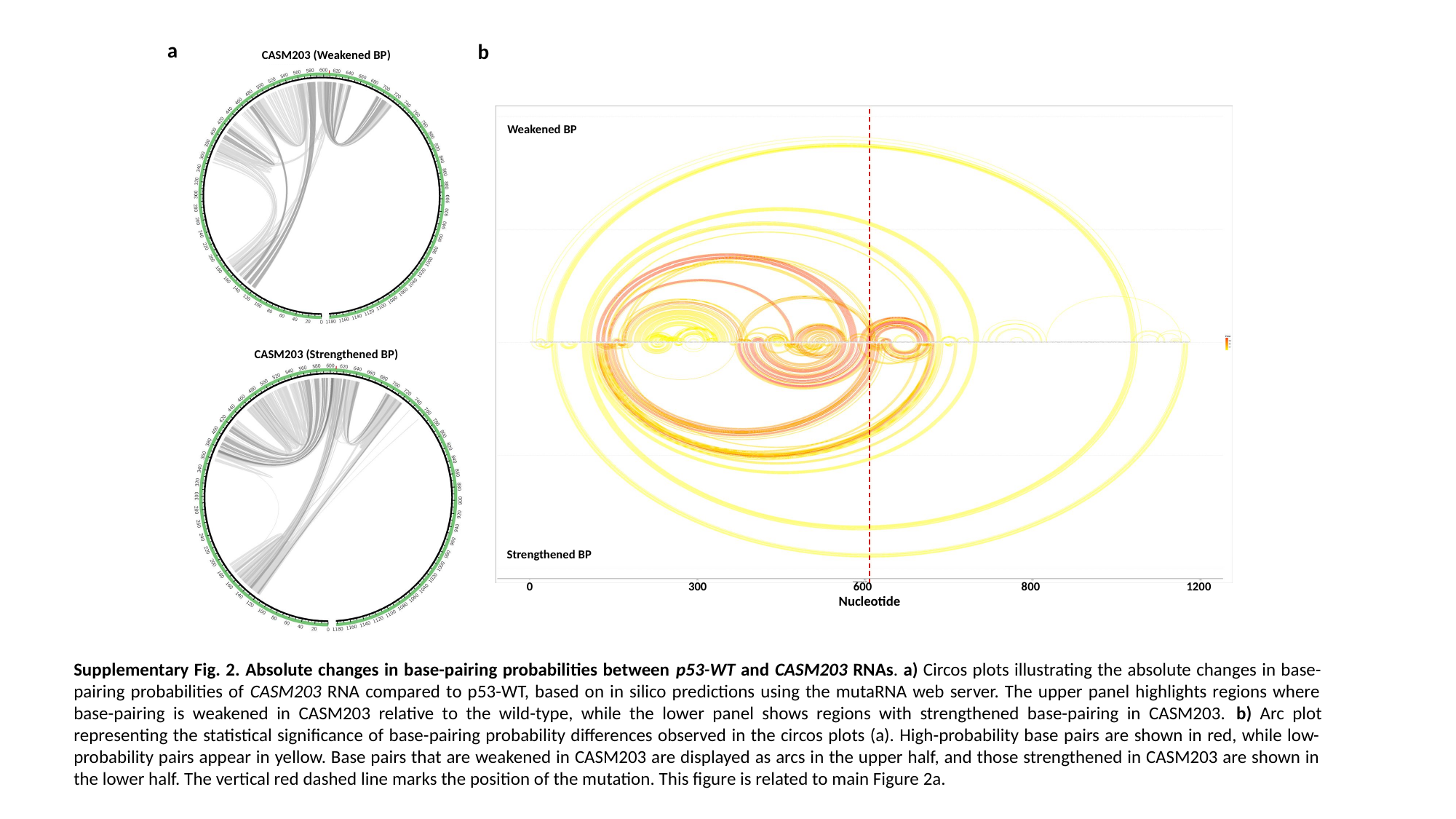

a
b
CASM203 (Weakened BP)
Weakened BP
Strengthened BP
0
300
600
800
1200
CASM203 (Strengthened BP)
Nucleotide
Supplementary Fig. 2. Absolute changes in base-pairing probabilities between p53-WT and CASM203 RNAs. a) Circos plots illustrating the absolute changes in base-pairing probabilities of CASM203 RNA compared to p53-WT, based on in silico predictions using the mutaRNA web server. The upper panel highlights regions where base-pairing is weakened in CASM203 relative to the wild-type, while the lower panel shows regions with strengthened base-pairing in CASM203. b) Arc plot representing the statistical significance of base-pairing probability differences observed in the circos plots (a). High-probability base pairs are shown in red, while low-probability pairs appear in yellow. Base pairs that are weakened in CASM203 are displayed as arcs in the upper half, and those strengthened in CASM203 are shown in the lower half. The vertical red dashed line marks the position of the mutation. This figure is related to main Figure 2a.

## Slide 3
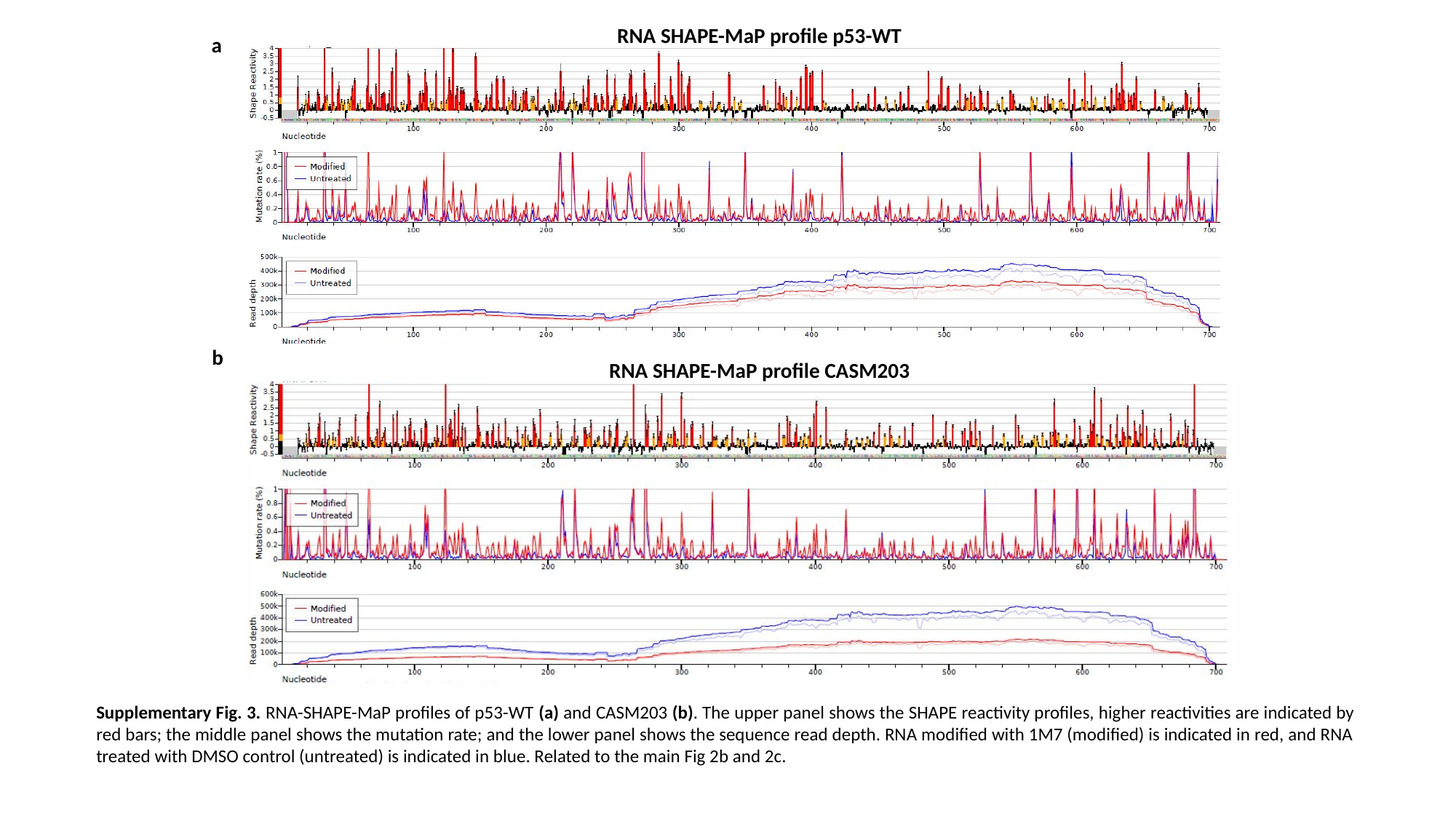

RNA SHAPE-MaP profile p53-WT
a
b
RNA SHAPE-MaP profile CASM203
Supplementary Fig. 3. RNA-SHAPE-MaP profiles of p53-WT (a) and CASM203 (b). The upper panel shows the SHAPE reactivity profiles, higher reactivities are indicated by red bars; the middle panel shows the mutation rate; and the lower panel shows the sequence read depth. RNA modified with 1M7 (modified) is indicated in red, and RNA treated with DMSO control (untreated) is indicated in blue. Related to the main Fig 2b and 2c.

## Slide 4
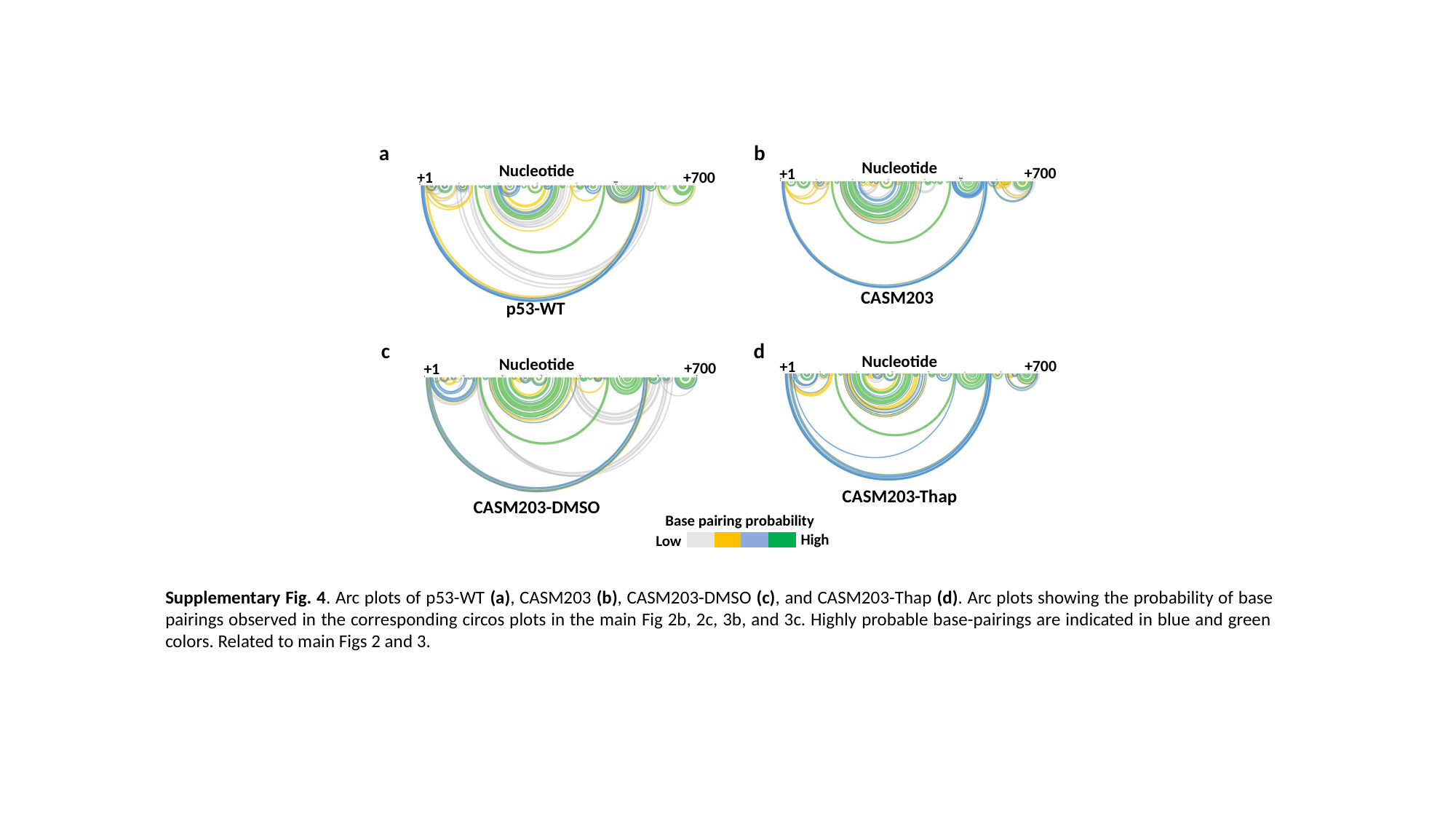

a
b
Nucleotide
Nucleotide
+700
+1
+1
+700
CASM203
p53-WT
c
d
Nucleotide
Nucleotide
+700
+1
+700
+1
CASM203-Thap
CASM203-DMSO
Base pairing probability
High
Low
Supplementary Fig. 4. Arc plots of p53-WT (a), CASM203 (b), CASM203-DMSO (c), and CASM203-Thap (d). Arc plots showing the probability of base pairings observed in the corresponding circos plots in the main Fig 2b, 2c, 3b, and 3c. Highly probable base-pairings are indicated in blue and green colors. Related to main Figs 2 and 3.

## Slide 5
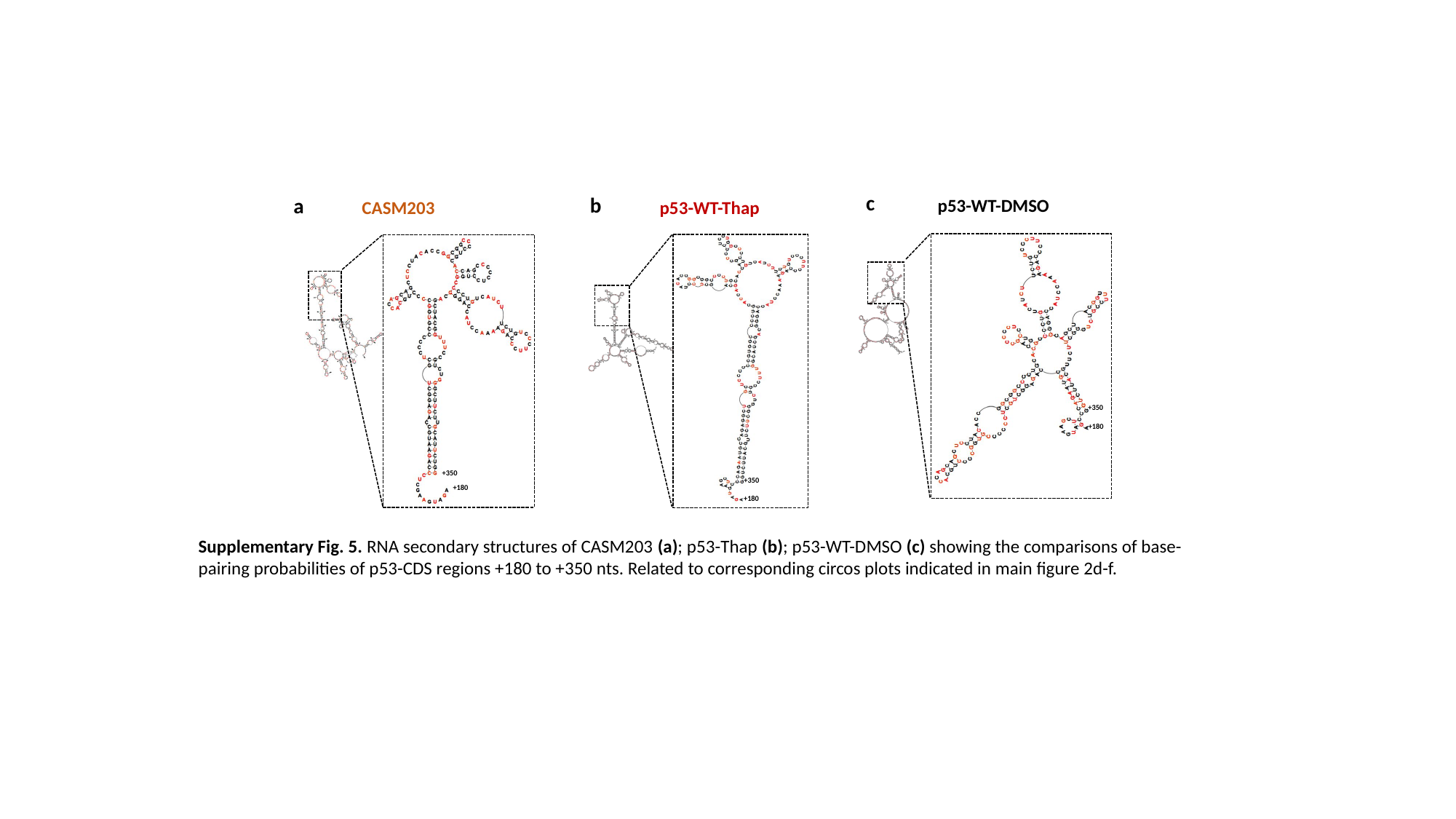

c
b
a
p53-WT-DMSO
p53-WT-Thap
CASM203
+350
+180
+350
+350
+180
+180
Supplementary Fig. 5. RNA secondary structures of CASM203 (a); p53-Thap (b); p53-WT-DMSO (c) showing the comparisons of base-pairing probabilities of p53-CDS regions +180 to +350 nts. Related to corresponding circos plots indicated in main figure 2d-f.

## Slide 6
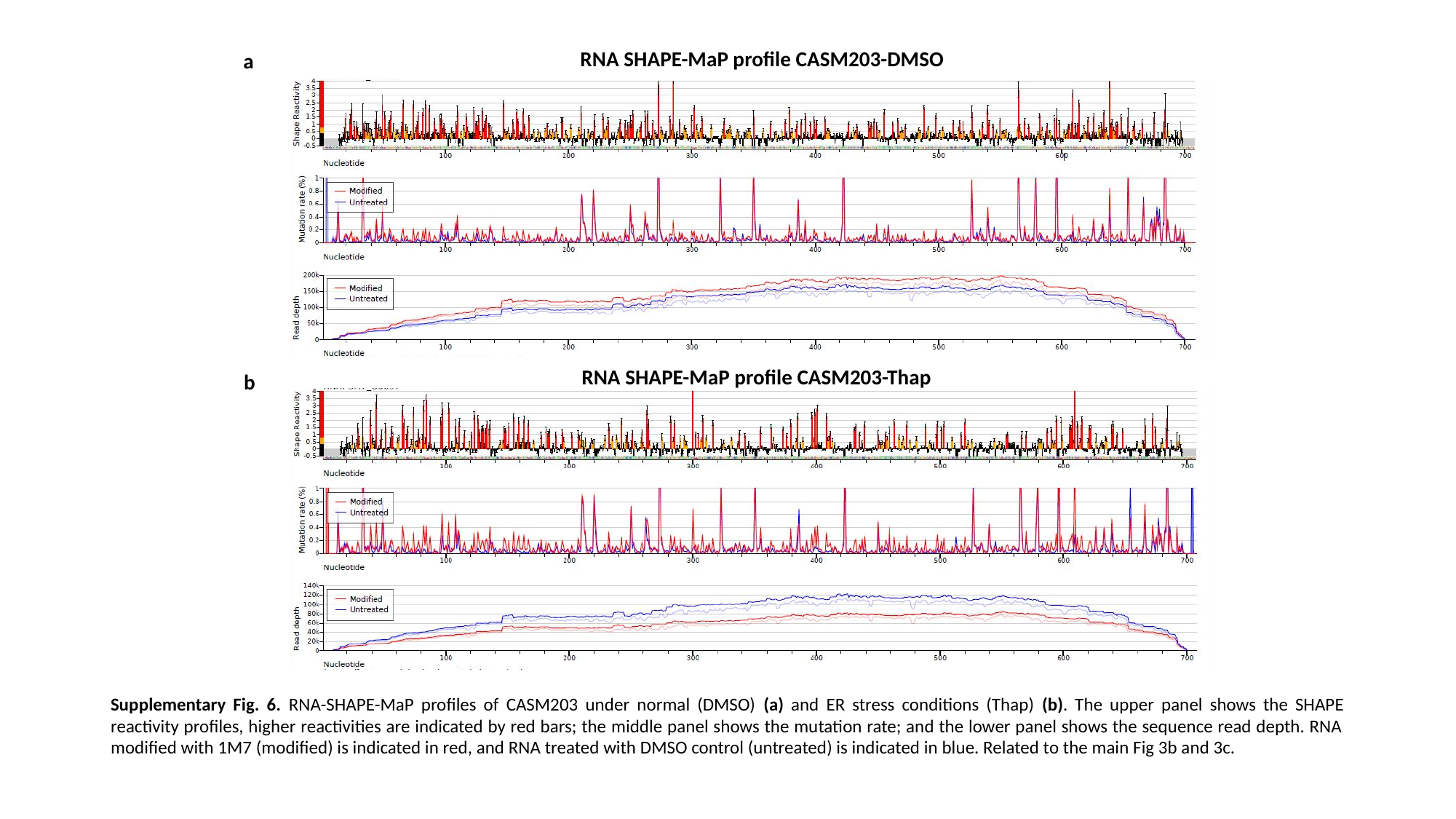

RNA SHAPE-MaP profile CASM203-DMSO
a
RNA SHAPE-MaP profile CASM203-Thap
b
Supplementary Fig. 6. RNA-SHAPE-MaP profiles of CASM203 under normal (DMSO) (a) and ER stress conditions (Thap) (b). The upper panel shows the SHAPE reactivity profiles, higher reactivities are indicated by red bars; the middle panel shows the mutation rate; and the lower panel shows the sequence read depth. RNA modified with 1M7 (modified) is indicated in red, and RNA treated with DMSO control (untreated) is indicated in blue. Related to the main Fig 3b and 3c.

## Slide 7
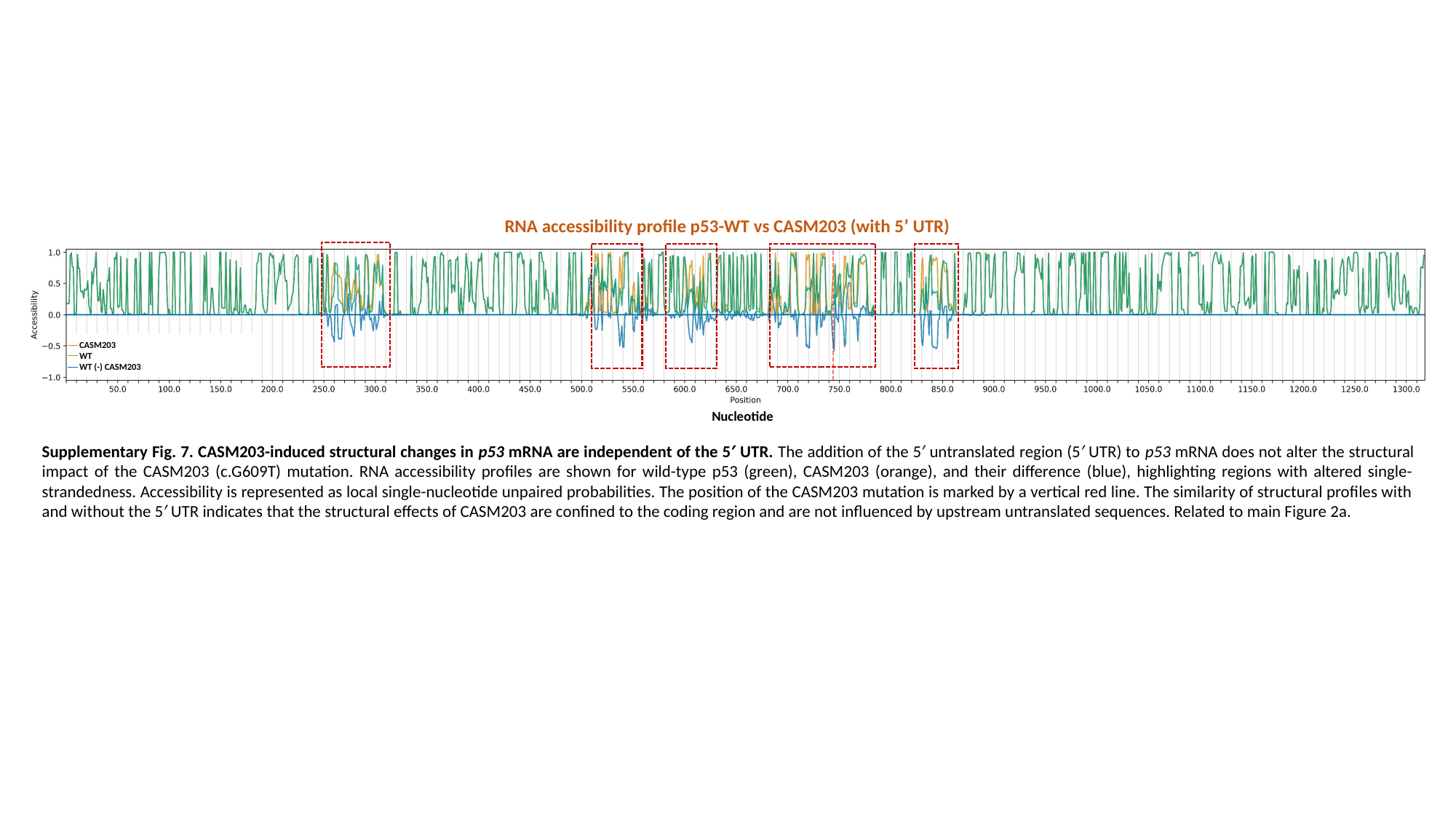

RNA accessibility profile p53-WT vs CASM203 (with 5’ UTR)
CASM203
WT
WT (-) CASM203
Nucleotide
Supplementary Fig. 7. CASM203-induced structural changes in p53 mRNA are independent of the 5′ UTR. The addition of the 5′ untranslated region (5′ UTR) to p53 mRNA does not alter the structural impact of the CASM203 (c.G609T) mutation. RNA accessibility profiles are shown for wild-type p53 (green), CASM203 (orange), and their difference (blue), highlighting regions with altered single-strandedness. Accessibility is represented as local single-nucleotide unpaired probabilities. The position of the CASM203 mutation is marked by a vertical red line. The similarity of structural profiles with and without the 5′ UTR indicates that the structural effects of CASM203 are confined to the coding region and are not influenced by upstream untranslated sequences. Related to main Figure 2a.

## Slide 8
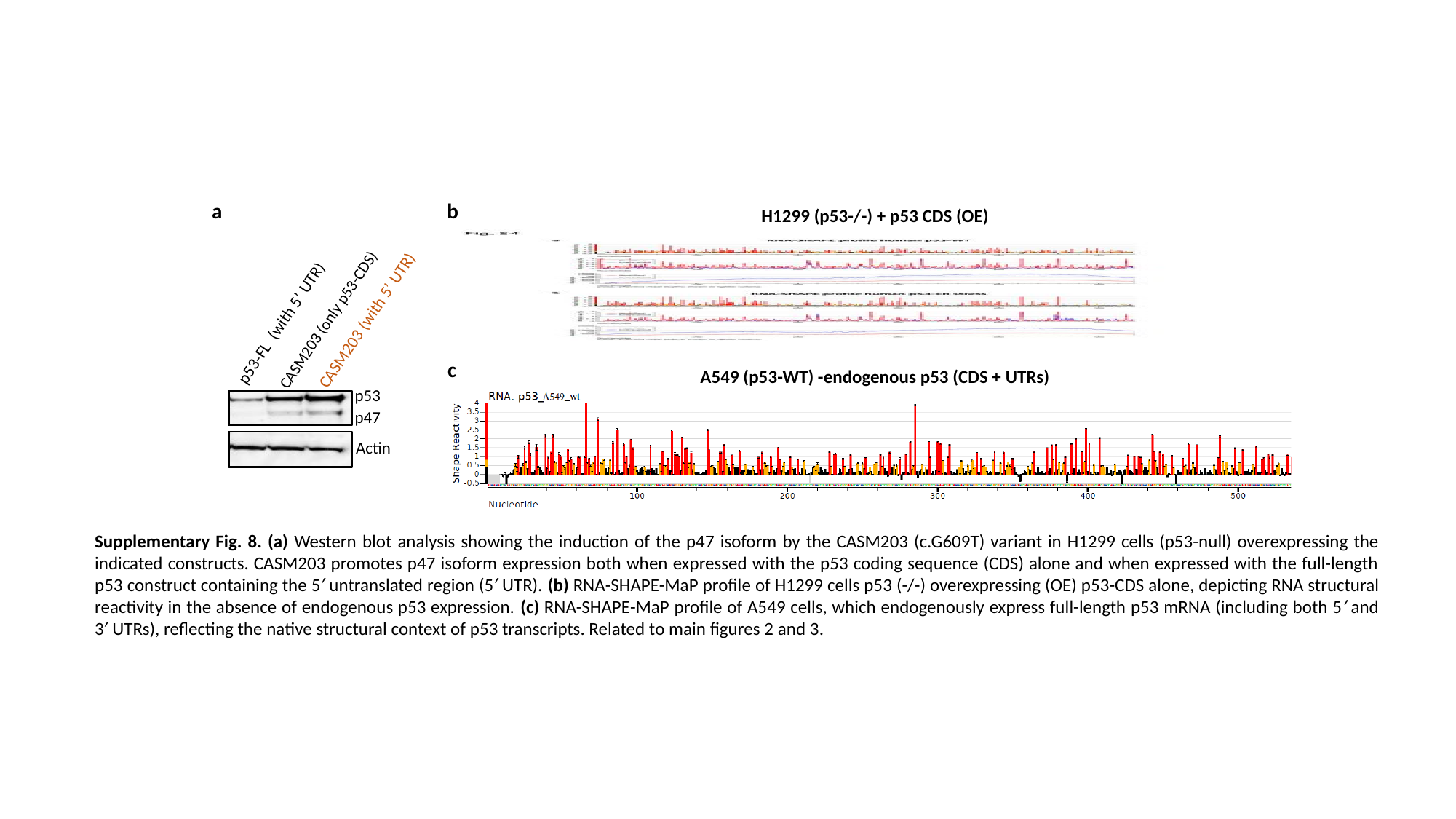

CASM203 (only p53-CDS)
CASM203 (with 5’ UTR)
p53-FL (with 5’ UTR)
p53
p47
Actin
a
b
H1299 (p53-/-) + p53 CDS (OE)
c
A549 (p53-WT) -endogenous p53 (CDS + UTRs)
Supplementary Fig. 8. (a) Western blot analysis showing the induction of the p47 isoform by the CASM203 (c.G609T) variant in H1299 cells (p53-null) overexpressing the indicated constructs. CASM203 promotes p47 isoform expression both when expressed with the p53 coding sequence (CDS) alone and when expressed with the full-length p53 construct containing the 5′ untranslated region (5′ UTR). (b) RNA-SHAPE-MaP profile of H1299 cells p53 (-/-) overexpressing (OE) p53-CDS alone, depicting RNA structural reactivity in the absence of endogenous p53 expression. (c) RNA-SHAPE-MaP profile of A549 cells, which endogenously express full-length p53 mRNA (including both 5′ and 3′ UTRs), reflecting the native structural context of p53 transcripts. Related to main figures 2 and 3.
