# Supplementary material for: A cancer-associated TP53 synonymous mutation induces synthesis of the p53 isoform p53/47
Source: Br J Cancer. 2025 Jul 26;133(7):970–5. doi: 10.1038/s41416-025-03127-w (PMC12480914; doi:10.1038/s41416-025-03127-w)
Supplement: Supplementary file 2 — Supplementary information_raw western blots [file 41416_2025_3127_MOESM2_ESM.pptx]

## Slide 1
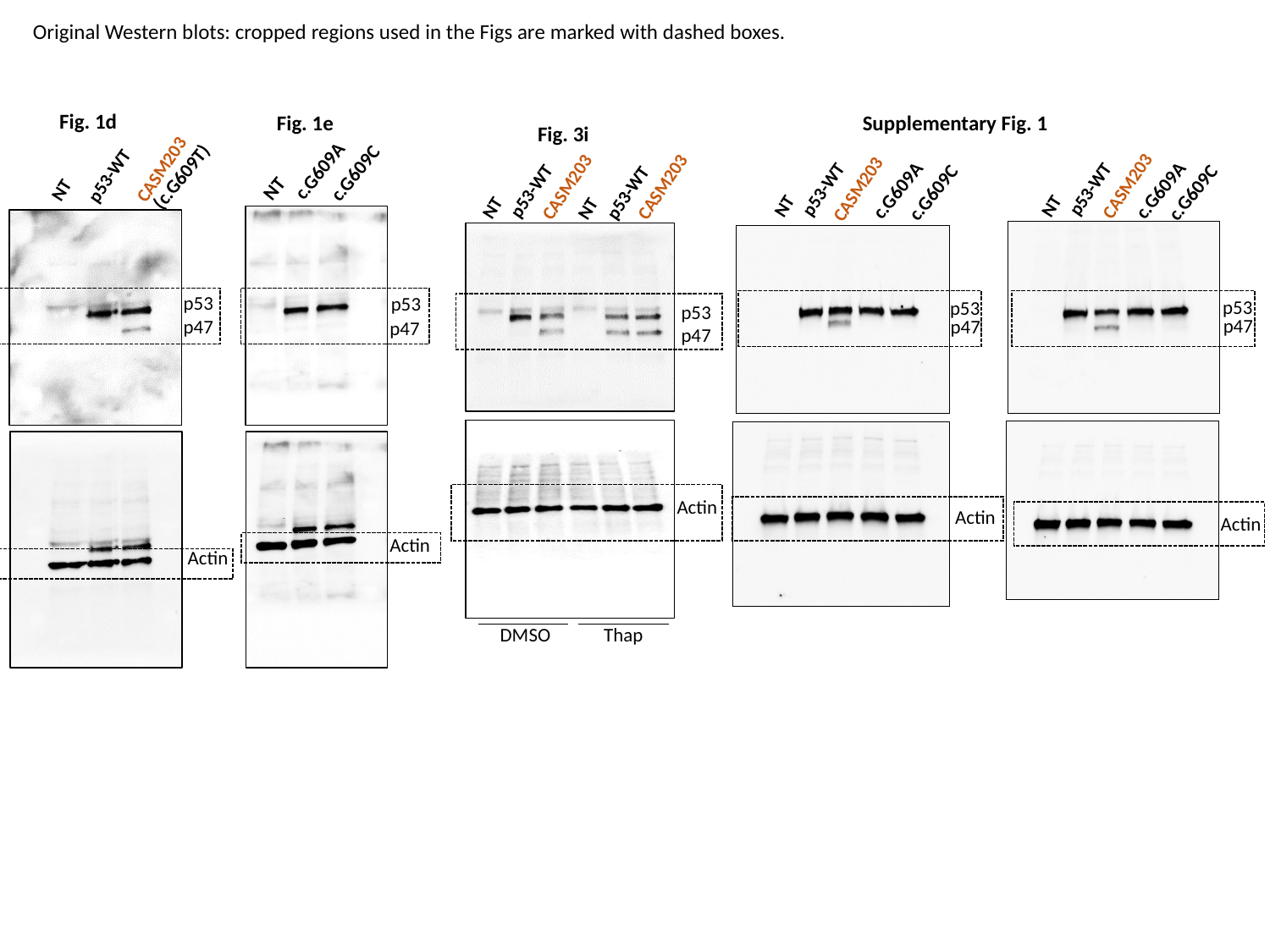

Original Western blots: cropped regions used in the Figs are marked with dashed boxes.
p53-WT
p53-WT
NT
NT
p53
p47
Actin
Thap
DMSO
CASM203
CASM203
Fig. 1d
Fig. 1e
Supplementary Fig. 1
Fig. 3i
CASM203
CASM203
CASM203
p53-WT
c.G609A
c.G609C
p53-WT
p53-WT
NT
(c.G609T)
NT
c.G609A
c.G609A
NT
NT
c.G609C
c.G609C
p53
p53
p53
p53
p47
p47
p47
p47
Actin
Actin
Actin
Actin

## Slide 2
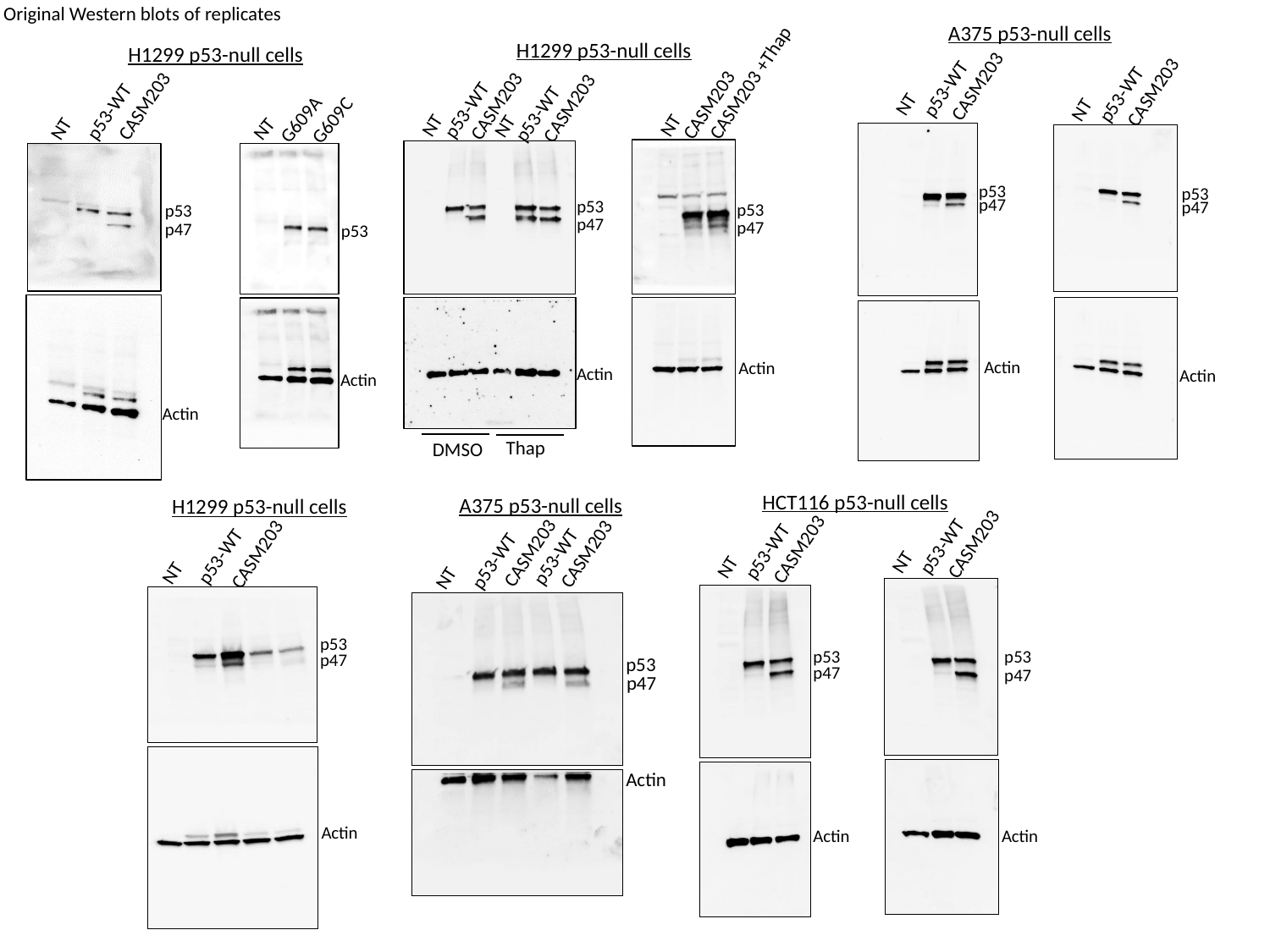

CASM203
p53-WT
NT
p53
p47
Actin
p53-WT
p53-WT
NT
NT
p53
p47
Actin
Thap
DMSO
CASM203
CASM203
Original Western blots of replicates
A375 p53-null cells
H1299 p53-null cells
CASM203
H1299 p53-null cells
CASM203
CASM203
CASM203 +Thap
p53-WT
p53-WT
NT
NT
G609A
G609C
NT
p53
Actin
NT
p53
p53
p47
p47
p53
p47
Actin
Actin
Actin
HCT116 p53-null cells
A375 p53-null cells
H1299 p53-null cells
CASM203
CASM203
CASM203
CASM203
CASM203
p53-WT
p53-WT
p53-WT
p53-WT
p53-WT
NT
NT
NT
NT
p53
p47
Actin
p53
p53
p53
p47
p47
p47
Actin
Actin
Actin
